# Advances in tea breeding in Japan: from traditional methods to genomic approaches

**DOI:** 10.1270/jsbbs.25050

**Published:** 2026-02-13

**Authors:** Yoshiki Ishiguro, Hiroto Yamashita, Takashi Ikka

**Affiliations:** 1 United Graduate School of Agricultural Science, Gifu University, 1-1 Yanagido, Gifu 501-1193, Japan; 2 Faculty of Agriculture, Shizuoka University, 836 Ohya, Suruga-ku, Shizuoka 422-8529, Japan; 3 Research Institute of Green Science and Technology, Shizuoka University, 836 Ohya, Suruga-ku, Shizuoka 422-8529, Japan; 4 Institute for Tea Science, Shizuoka University, 836 Ohya, Suruga-ku, Shizuoka 422-8529, Japan

**Keywords:** tea plant, genomic prediction, genomic selection, marker-assisted selection, germplasm

## Abstract

Tea, made from the tea plant (*Camellia sinensis* L.), is one of the most widely consumed beverages worldwide. Traditional breeding methods have contributed to the development of cultivars with desirable traits; however, these breeding approaches are time-consuming and constrained by the limited genetic diversity of tea plants. Despite the significant impact of genomic information on crop breeding, its application in tea plants has remained limited. Recently, the rapid accumulation of genomic resources for tea plants has enabled the research development for markers-assisted selection and genomic selection in tea breeding. These genomics-based approaches are positioned as complementary methods to phenotypic selection. In this review, we summarize the advancements in breeding technologies for tea in Japan and highlight future directions for genomics-based tea breeding.

## Introduction

Tea (*Camellia sinensis* L.) is a woody plant in the Theaceae family of angiosperms. It is mainly cultivated in China, Japan, and India, and can be broadly categorized into two types; the Chinese type (*C. sinensis* var. *sinensis*), which has small leaves and strong freezing resistance, and the assam type (*C. sinensis* var. *assamica*), which has large leaves and weak freezing resistance (Banerjee 1992, Kaundun and Matsumoto 2003). Tea contains functional metabolites such as flavonoids, free amino acids, and caffeine. Catechins are major constituents of tea flavonoids, and mainly consist of (–)-epicatechin (EC), (–)-epicatechin gallate (ECG), (–)-epigallocatechin (EGC), and (–)-epigallocatechin gallate (EGCG) (Reygaert 2018). The most abundant catechin in tea is EGCG, which is beneficial for human health because of its antioxidant (Feng *et al.* 2013) and anti-inflammatory (Trompezinski *et al.* 2003) properties. Theanine is an amino acid that is found in certain species of the fungus *Xerocomus*, and in *C. sinensis*, *Camellia japonica*, and *Camellia sasanqua* (Juneja *et al.* 1999). Theanine contributes to the umami taste of tea (Narukawa *et al.* 2008). Caffeine has various effects on human health, most notably a stimulant effect. It naturally occurs in some foods such as tea, coffee, and cocoa (Ashihara and Crozier 2001, Gramza-Michałowska 2014).

Tea is one of the most popular beverages worldwide (FAO 2022). In recent years, tea exports from Japan have increased because of the Japanese food boom and growing health consciousness in the U.S.A. and other countries (Qin and Zhou 2024). Whereas tea production has been increasing on a global scale, tea production in Japan has been decreasing annually (MAFF 2025). In addition, the bias toward certain cultivars in tea plantations has led to uniformity of quality, excessive labor demands, and increased risk of weather-related disasters. Although the introgression of novel alleles into the tea germplasm by breeding is slow and tedious, it is a key requirement to increase the genetic diversity of tea resources to maintain and enhance tea production and to overcome the bias towards a single cultivar (Tester and Langridge 2010).

Conventional breeding methods have produced many representative tea cultivars in Japan and other countries; but these approaches are time-consuming and costly. Next generation sequencing (NGS) technology has provided new genetic information for tea breeders, and has provided an opportunity to transition from conventional to genomics-assisted breeding. To recover tea production, meet various needs through breeding, and promote tea research in the future, it is necessary to review information on tea breeding from the past to the present.

In this review, we summarize the evolution of tea breeding from traditional approaches to modern methodologies, highlighting recent advances that utilize DNA markers. Furthermore, we discuss future perspectives on genomics-based breeding strategies for tea plants.

## Traditional tea breeding in Japan

Tea breeding started in the late 1800’s in Japan (Takeda 2004). In the beginning, private breeders attempted to develop tea cultivars. But, propagating tea plants from seed proved challenging due to their allogamous nature with high heterozygosity; that is, they require cross-pollination for successful seed set. A major breakthrough came with Hikosaburo Sugiyama, one of most famous private tea plant breeders, who discovered the Japanese elite cultivar ‘Yabukita’. He also played a key role in advancing vegetative propagation techniques (Akino 1973). Systematic tea breeding began in the early 1900s, with segregation breeding becoming the major method (Tanaka 2012). Hybrid breeding was initiated in 1932, initially targeting the development of black tea cultivars. In 1935, the introduction of propagation by cuttings facilitated the widespread distribution of improved cultivars (Takeda 2007). In particular, ‘Yabukita’ has excellent rooting ability for cuttings and contributed to the spread of tea.

**Fig. 1.**
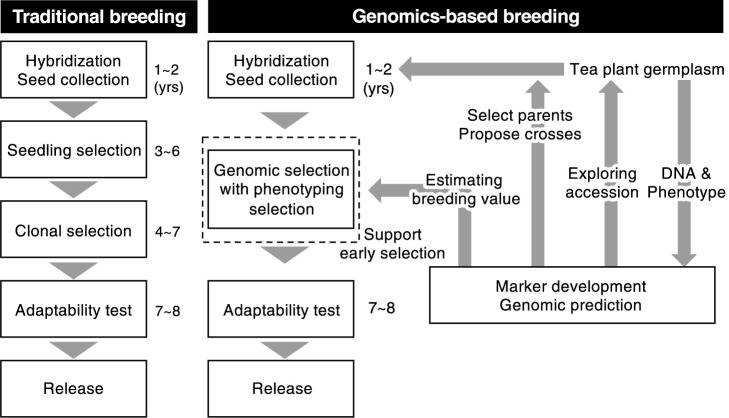
Schematic representation of a genomic selection (GS)-based tea breeding pipeline. GS supports early prioritization of candidates, parental selection, and crossing design. Seedling and clonal phenotyping remain essential, and adaptability testing is initiated only after multi-year, multi-environment evaluations confirm performance and stability.

Today, crossbreeding and propagation form the basis of tea breeding. However, the development of new cultivars through crossbreeding remains a time-consuming process. According to the tea breeding guidelines revised in 1999, the entire breeding cycle typically spans over 25 years as follows (Fig. 1): the guidelines outline 1–2 years for hybridization and initial selection, 3–6 years for individual growth and selection, 1–2 years for breeding and selection of vegetatively propagated individuals, 4–7 years for comparative trials, and 7–8 years for adaptability testing. On average, it takes approximately 25 years from the initiation of breeding to cultivar registration (Takeda 2007).

## Development and impacts of tea cultivars in Japan

Among the tea cultivars grown in Japan, ‘Yabukita’ accounts for the largest proportion of the total tea cultivation area (62.2%), followed by ‘Yutakamidori’ (6.0%), ‘Saemidori’ (4.4%), ‘Okumidori’ (3.4%), ‘Sayamakaori’ (2.0%), and ‘Samidori’ (0.7%) (MAFF 2025). ‘Yabukita’, registered in 1953, is a leading cultivar in the Japanese tea industry. Although ‘Yabukita’ has superior quality and agronomic traits compared to other cultivars, it also has a weakness in its sensitivity to anthracnose and gray blight. It has played as significant role as a breeding parent; however, its extensive use has led to a reduction in the genetic diversity of the tea germplasm in Japan (Yamashita *et al.* 2019). ‘Yutakamidori’ was selected from a seedling of ‘Asatsuyu’. It has earlier bud sprouting than ‘Yabukita’, and is cultivated in southern parts of Japan, such as Kagoshima Prefecture. ‘Saemidori’ and ‘Okumidori’ were developed to address the labor challenges caused by the dominance of ‘Yabukita’. ‘Saemidori’ is a hybrid of ‘Yabukita’ and ‘Asatsuyu’, and was registered in 1991. It exhibits early bud sprouting and a high amino acid content. Among the cultivars developed through crossbreeding, it is the most widely cultivated and is considered one of the iconic cultivars in Japanese tea breeding (Kawahara *et al.* 2024). ‘Okumidori’ is a hybrid of ‘Yabukita’ and ‘Shizuoka landrace No.16’. Due to its later bud sprouting, which helps reduce labor challenges, it is expected to serve as a suitable replacement for ‘Yabukita’ in replanting of aging tea plantations.

To promote tea consumption, breeders have developed cultivars with unique functional components and coloration. ‘Sun Rouge’ is one of the most notable tea cultivars in Japan due to its purple leaves, which contain a high level of anthocyanins (Fujimura *et al.* 2011). It was selected from a seedling of ‘Cha Chuukanbohon Nou 6’, which was derived from a cross between *Camellia taliensis* and *C. sinensis*. The albino tea cultivars with chlorotic or etiolated leaves (i.e., white or yellow coloration, respectively), such as ‘Koganemidori’ and ‘Kiraka’, are rich in free amino acids (Morita *et al.* 2011, Yamashita *et al.* 2021a). Rising consumer interest in the health-promoting functions of tea has also highlighted the need for cultivars with enhanced nutraceutical properties. ‘Benifuuki’ contains a high concentration of epigallocatechin-3-*O*-(3-*O*-methyl) gallate (EGCG3ʹMe), a compound known for its anti-obesity and antiallergic effects (Oritani *et al.* 2009, Sano *et al.* 1999). ‘Sofu’ is a rare cultivar that contains quercetin, a polyphenol with potent and cholesterol-lowering (Kondo *et al.* 2003). In recent years, the popularity of *matcha* has increased, leading to a growing demand for cultivars suitable for these processing styles (Yoshida *et al.* 2018). Compared with ‘Yabukita’ and ‘Saemidori’, the cultivar ‘Seimei’ has a lower catechin content and a higher amino acid content (Kawahara *et al.* 2024), making it particularly suitable for *matcha* (Yoshida *et al.* 2018). These cultivars illustrate how health science is increasingly shaping breeding targets, shifting priorities from conventional goals such as yield and processing quality toward the enrichment of functional compounds. Such breeding strategies are in line with evolving societal demands and emphasize the potential of genomics-assisted breeding to accelerate the development of health-oriented tea cultivars.

In summary, representative Japanese tea cultivars have been developed through traditional breeding methods, reflecting the remarkable skill and efforts of breeders. Over time, a wide range of cultivars has been produced, with the primary breeding strategy shifting from individual selection to crossbreeding. Genomics-based approaches now offer powerful tools to meet evolving health-related demands by enabling the development of cultivars enriched with specific functional compounds.

## Accelerating tea breeding with genomic information

### Tea breeding using genomic information

Although crop breeders have developed a wide range of cultivars through crossbreeding, this approach is time-consuming because tea plants are perennial woody plants with a long-life cycle and large canopy area. Consequently, genomics-based breeding represents a promising complementary approach. Marker-assisted selection (MAS) and genomic selection (GS) are two representative genomics-based breeding methods, and their application in tea breeding has been investigated. In this section, we summarize research on genomics-based approaches for tea breeding.

### Marker-assisted breeding

MAS is based on DNA markers within quantitative trait loci (QTLs) or in regions of linkage disequilibrium (LD) with the QTL (Mauricio 2001). Identifying QTLs governing quantitative traits requires constructing genetic maps based on recombination rates, followed by subsequent QTL analysis. High density linkage maps facilitate more accurate recombination rate estimation, and such maps have been successfully constructed and applied to QTL analysis (Hackett *et al.* 2000, Taniguchi *et al.* 2012, Xu *et al.* 2018). These approaches have been applied in tea breeding to target traits such as disease resistance, leaf coloration, and functional components. For example, MAS has been used to develop tea cultivars with enhanced resistance to mulberry scale, a pest that is difficult to control and causes severe damage to tea plants. With an overall goal to develop resistant cultivars, Tanaka (2006) developed random amplified polymorphic DNA (RAPD) markers associated with mulberry scale using a cross population derived from ‘Sayamakaori’, a mulberry scale-resistant cultivar, and ‘Kanaya-Ck17’ (Tanaka 2006). These markers were reliably found in the resistant individuals in the F_1_ population. Subsequently, breeders have utilized these markers to develop additional mulberry scale resistant cultivars such as ‘Nanmei’ (Taniguchi *et al.* 2018), ‘Kanaemaru’ (Saba *et al.* 2021), and ‘Danshin 37’ (Yoshidome *et al.* 2022). Blister blight, caused by the obligate fungal pathogen *Exobasidium vexans* Masse (Basidiomycetes), remains one of the most devastating foliar diseases of tea. Karunarathna *et al.* (2021) developed an effective marker for resistance to blister blight leaf disease based on expressed sequence tag (Karunarathna *et al.* 2021). Wang *et al.* (2010) developed a RAPD marker that distinguished the albino cultivars ‘Qiannianxue’ and ‘Xiaoxueya’, but was absent in the non-albino cultivar ‘Fudingdabai’ (Wang *et al.* 2010). This marker could reliably identify whether a cultivar was an albino or not. More recently, Xie *et al.* (2025) conducted a pangenome analysis of various tea accessions, including the purple-leaf tea cultivar ‘Zijuan’, and identified a 181-bp transposable element (TE) located in the promoter region of *CsMYB75*, which is present in purple-leaf tea (Xie *et al.* 2025). This TE was shown to regulate the expression of *CsMYB75*, which encodes a transcription factor that regulates anthocyanin biosynthesis; this TE was detected in bright-purple accessions but was absent in green or light-purple accessions. Therefore, this TE represents a promising DNA marker for anthocyanin content in MAS applications.

Ogino *et al.* (2019) identified a caffeine-less individual among the progeny of a natural cross between ‘Cha Chuukanbohon Nou 6’ and *C. sinensis* (*Camellia taliensis* × *C. sinensis*). A progeny analysis suggested that the caffeine-less trait was controlled by a single recessive locus, which was attributed to an allele derived from ‘Taliensis-akame’, the seed parent of ‘Cha Chuukanbohon Nou 6’. Later, Ogino *et al.* (2019) investigated the sequence of tea caffeine synthase 1 (TCS1), a gene strongly associated with the caffeine-less trait, and conducted a polymorphism analysis of the *TCS1* gene in ‘Cha Chuukanbohon Nou 6’, ‘Makura F1-95180’, and 101 individuals from their cross population (Ogino *et al.* 2019). They identified three insertions in the *TCS1* gene and developed DNA markers accordingly. These markers were present in the caffeine-less F_1_ hybrids and ‘Yabukita’, confirming their suitability for identifying caffeine-less tea plants in MAS applications. Similarly, Zhong *et al.* (2022) constructed a hybrid population from ‘Ruyuan Kucha’ and ‘Zhongcha 302’, and observed a negative correlation between the contents of caffeine and theacrine, which is a unique purine alkaloid converted from caffeine in the progeny (Zhong *et al.* 2022). Bulked segregant RNA-seq analyses revealed that the transcript level of the gene encoding theacrine synthase was positively associated with theacrine content. Furthermore, they developed a 604-bp InDel marker based on 271 bp insert in promoter region of *theacrine synthase* (*TcS*), leading to distinguishing high-theacrine and low-caffeine accessions.

Collectively, these studies highlight the applications of MAS in tea breeding. While MAS is highly effective for selecting qualitative traits associated with the presence or absence of specific markers, its application to quantitative traits controlled by multiple genes remains limited. Genomic prediction (GP) based on genome-wide DNA markers offers a promising approach to solving these challenges.

### Genomic selection/Genomic prediction

Genomic prediction builds statistical models to predict genomic estimated breeding values (GEBVs) using genome-wide DNA markers in LD with their QTLs, thereby enabling breeders to estimate the potential for quantitative traits (Meuwissen *et al.* 2001). One of the key advantages of GP is its ability to leverage large datasets, including germplasm and historical breeding data. Selection based on GP is called genomic selection (GS) and has been widely applied in many crops. For example, GS has been applied to predict fruit quality in apple (Kumar *et al.* 2012), citrus (Minamikawa *et al.* 2017), and strawberry (Yamamoto *et al.* 2021), and seed yield in buckwheat (Yabe *et al.* 2018).

Several studies have evaluated the potential of GP in tea populations. Koech *et al.* (2020) applied GP to estimate quality-related and drought tolerance traits in black tea using 1,421 diversity array technology sequencing (DArT-seq) markers from 255 F_1_ accessions (Koech *et al.* 2020). The prediction accuracy (percentage accordance) was moderate for relative water content (%RWC) (54.4%–68.5%) and catechin content (54.0%–68.7%), and high for caffeine content (56.0%–82.1%), aroma (50.5%–100%), astringency (51.9%–93.3%), brightness (51.0%–79.0%), briskness (57.6%–80.0%), and color (51.0%–58.6%). Yamashita *et al.* (2020b) also performed GP for tea quality-related traits and color using 9,523 double-digest restriction site-associated DNA sequencing (ddRAD-seq) markers identified from 150 accessions, mainly Japanese cultivars and landraces (Yamashita *et al.* 2020b). This study achieved moderate prediction accuracy for EC content (*r* = 0.17–0.28), ECG content (*r* = 0.25–0.31), EGCG content (*r* = 0.32–0.41), total catechin content (*r* = 0.27–0.32), and caffeine content (*r* = 0.44–0.51), but low accuracies for amino acids, including theanine. The authors attributed these results to the small population size and/or low heritability of the traits. Lubanga *et al.* (2021) applied GP to predict tea quality traits such as free amino acid content, polyphenol content, and alkaloid content using 2,779 single nucleotide polymorphisms (SNPs) obtained through genotyping-by-sequencing (GBS) of 103 accessions (Lubanga *et al.* 2021). Prediction accuracy was high for ECG content (*r* = 0.56), theogallin content (*r* = 0.59), and theobromine content (*r* = 0.61), but low for theanine content. The authors highlighted the potential of GS in tea breeding and suggested that early application at the seedling stage could offer significant benefits.

Several studies have demonstrated the potential of GP for tea agronomic traits and quality-related traits; however, several challenges remain to be addressed: (1) For some traits, the prediction accuracy could be improved by optimizing the training population; (2) prediction models should be validated using independent populations, such as hybrid population or germplasm; and (3) the range of target traits should be expanded to include not only agronomic traits but also even more quality-related traits and those related to adaptability under changing environmental conditions. In addition, the strategy for implementing GP in tea breeding is a critical consideration. Future studies that integrate epistasis-aware models with large-scale, multi-environment trials across diverse climates and management practices will be essential for fully realizing the potential of GP in tea breeding.

### Reference genome information

Reference genomes are essential for analyses of germplasm genetic diversity and for developing breeding tools. To date, more than 30 high-quality tea plant genomes have been published (Table 1). In the new genome assembly, sequencing results and assembly quality are generally evaluated based on metrics such as N50 and benchmarking universal single-copy orthologs (BUSCO). N50 is the length for which sequences of length ≥ L account for 50% of the assembly: BUSCO reports the proportion of expected single copy orthologs that are complete, fragmented, or missing (Simão *et al.* 2015). The first draft genome of *C. sinensis* var. *assamica* cv. ‘Yunkang10’, reported in 2017, was of low quality with a scaffold N50 of 0.45 Mb and a BUSCO score of only 85% (Xia *et al.* 2017). Wei *et al.* (2018) released the first chromosome-scale genome assembly for var. *sinensis*, cv. ‘Shuchazao’, which had a size of 2.94 Gb, compared to 2.89 Gb for the draft genome (Wei *et al.* 2018, Xia *et al.* 2020). In 2020, ‘Longjing 43’, which is among the most widely cultivated tea cultivar in China, was reported (Wang *et al.* 2020). They showed that it had the largest genome size of any tea cultivar reported to date and it exhibited BUSCO scores of 88.36%. Zhang *et al.* (2021) reported the first haplotype-resolved genome assembly of var. *sinensis* cv. ‘Tieguanyin’ using their developed algorithms (Zhang *et al.* 2019, 2021). They showed ‘Tieguanyin’ maintained high levels of coding sequence similarity, and identified large-effect allelic variations that may influence gene function. More recently, genome assembly of var. *assamica* cv. ‘Zijuan’, var. *sinensis* cv. ‘Anjibaicha’, and wild tea plant ‘L618’ have been reported, all exhibiting higher completeness with BUSCO scores of 98.93%, 99.14%, and 99.05%, respectively, regardless of their ancestry (Tariq *et al.* 2024). These genome assemblies provide valuable references for GP and genome-wide association study (GWAS). The BUSCO score alone does not provide a comprehensive assessment of genome assembly quality. Instead, it is essential to consider the intended applications of genomic data. In addition to BUSCO, other evaluation metrics, such as the LTR assembly index (LAI), which assesses genome completeness and structural accuracy among closely related species or cultivars, should be utilized (Ou *et al.* 2018). Integrating multiple metrics is particularly critical for genome assemblies intended for breeding applications.

Previous studies reported genetic differences between Japanese and exotic tea accessions (Matsumoto *et al.* 2004, Taniguchi *et al.* 2014), but the genome assembly of Japanese tea accessions remained poorly characterized. More recently, Kawahara *et al.* (2024) released the first chromosome-scale genome assembly of a Japanese green-tea cultivar, ‘Seimei’. The assembly exhibited a scaffold N50 of 203.00 Mb and a BUSCO score of 94.80% (Kawahara *et al.* 2024); these quality scores were comparable to those of ‘Huangdan’, which has been used as a reference genome in several studies (Tariq *et al.* 2024, Wang *et al.* 2021). Population genetics analysis based on this Japanese tea genome-assembly further revealed that human selection pressure has shaped the genetic diversity in genes such as *PPO* and *TCS1* in Japanese tea cultivars.

These reference genomes are easily accessible through public databases such as Tea Plant Information Archive for *Camellia* genomics (TPIA2, http://tpia.teaplants.cn) and TASUKE+ (https://agrigenome.dna.affrc.go.jp/tasuke/Tea_Seimei) (Gao *et al.* 2024, Kawahara *et al.* 2024). Today, researchers and breeders can easily share and utilize genomic information.

## Perspectives for genomics-based breeding in tea plants

Traditional tea breeding primarily relies on experience-based selection to select the most suitable parental accessions and identify individuals with desirable traits among the progenies. Genomics-based breeding can serve as a complementary approach to conventional crossbreeding. The advent of the genomic era is expected to accelerate the adoption of genomic breeding strategies in tea plants. Several breeding-related technologies have been developed, and their future prospects are outlined below.

### Breeding of cultivars for climate change adaptation

Climate change is expected to negatively impact tea cultivation by increasing the risk of crop damage caused by fungal pathogens and various environmental stresses. Boehm *et al.* (2016) analyzed the relationship between tea yield and monsoon dynamics and reported that increased precipitation or decreased solar radiation negatively affects tea production (Boehm *et al.* 2016). Similarly, Pandey *et al.* (2021) reported that climate change adversely influences harvest timing and yield. These findings highlight the urgent need to develop tea cultivars and cultivation practices that are resilient to climate change (Pandey *et al.* 2021). Among the major threats, anthracnose, caused by fungi of the genus *Colletotrichum*, and gray blight, caused by *Pestalotiopsis longiseta* (Speg.) or *Pestalotiopsis theae* (Sawada) Steyaert, can significantly reduce tea yield. The frequency and severity of these diseases are expected to increase under future climate scenarios (Pandey *et al.* 2021). However, no major DNA markers for resistance to these diseases have been identified to date. Recently, a QTL analysis identified a candidate QTL associated with anthracnose resistance, which may facilitate the development of DNA markers for breeding disease-resistant cultivars (Zhang *et al.* 2025).

Advances in sequencing technologies have provided tea breeders with high-quality genomic data and new opportunities to identify DNA markers for breeding. Several studies have demonstrated that structural variants (SVs) play important roles in crop growth and adaptation. For example, SVs have been linked to stress tolerance in maize (Maron *et al.* 2013), soybean (Bayless *et al.* 2019), and oilseed (Gabur *et al.* 2020). In tea plants, a comparative genomic analysis of multiple cultivars has revealed SVs and copy number variants associated with catechin content, with specific variant combinations serving as potential DNA markers (Tariq *et al.* 2024). Further research is expected to identify SVs related to stress tolerance in tea plants and to facilitate the development of DNA markers for breeding climate-resilient, such as drought and high-temperature cultivars.

### Implementing GP/GS for tea breeding

Compared with MAS, implementing GP in tea breeding has progressed more slowly due to several challenges. In practice, GP accuracy and utility vary across traits. Low heritability and environmentally sensitive traits (e.g., yield and several quality attributes) remain challenging. Moreover, multi trait GP and economic index-based selection are needed to balance yield, quality, disease resistance, and environmental tolerance; however, robust, validated pipelines integrating all practically important traits are not yet available in tea. It is crucial to consider that qualitative traits such as leaf color are often more efficiently controlled by MAS than by GP, and traits influenced by the environment are also challenging. Factors such as low heritability, small population sizes, and low marker density reduce the prediction accuracy of GP, making its improvement a key focus for breeders. The impact of heritability on GP accuracy has been widely discussed in other crops (Ornella *et al.* 2012, Zhang *et al.* 2017), and similar analyses are needed for tea, given that inheritance patterns vary among traits. Low heritability can be mitigated through optimized phenotyping strategies and cultivation trials under controlled environments, while low marker density can be addressed by expanding marker numbers via whole-genome sequencing of larger-scale populations. Additionally empirical validation to assess prediction accuracy for untested accessions is essential for effectiveness (Yu *et al.* 2016). Optimizing the training population is another critical factor, as the genetic relatedness between the training and validation sets significantly influences GP accuracy (Akdemir *et al.* 2015, Rincent *et al.* 2012).

The development of effective breeding strategies incorporating GS has the potential to accelerate tea breeding. Lubanga *et al.* (2021) demonstrated that GS models trained on historical data can be applied at the nursery stage (Lubanga *et al.* 2021). Under conventional tea breeding programs, it typically takes about 15 years to reach the field trial phase. In contrast, genomics-based breeding evaluates traits directly from genotypes, enabling rapid identification of candidates based on quality and agronomic traits. Although GS is not yet able to replace individual or line selection in practical tea breeding, GS can be utilized to pre-select parental lines at early generations or to reduce the number of lines subjected to expensive and time-consuming phenotypic evaluations. While comprehensive field trials evaluating yield, quality, and stress resistance remain indispensable, GP can assist in identifying promising candidates in early phases, thereby contributing to breeding efficiency (Fig. 1). Future efforts are anticipated to lead to the proposal of a local genomic breeding model for tea that utilizes GP to enable the early evaluation of some breeding traits, thereby reducing the breeding cycle. This is subject to overcoming challenges such as broadening the range of applicable traits and enhancing prediction accuracy. This integration of GP and phenotypic selection is currently being explored in various crops and should be considered a realistic and synergistic approach for tea breeding, as it also facilitates the characterization of untested germplasm and supports a more diverse and efficient selection of breeding parents.

Recently, researchers have also focused on predicting heterosis in F_1_ populations. Heterosis refers to the phenomenon where F_1_ individuals exhibit superior phenotypes compared to their parents, resulting from dominant, overdominant, and epistatic effects. Predicting heterosis is particularly valuable for crops such as tea plants, which rely on F_1_ hybrids in breeding programs. Liu *et al.* (2022) developed a prediction model for cucumbers that incorporated dominance and non-additive effects, and demonstrated its effectiveness in predicting heterosis (Liu *et al.* 2022). While the genetic basis of heterosis in tea plant has been explored in a few studies (Wang *et al.* 2022), no prediction models have yet been developed. Sallam *et al.* (2020) have reported that applying haplotypes in the GS model improves prediction accuracy (Sallam *et al.* 2020). Constructing haplotype-resolved genomes allows for further analysis to reveal that relationship between haplotypes and traits, which could then be used to develop more accurate prediction models. Improving our understanding of trait inheritance in tea, combined with advanced statistical modeling, will enhance the potential of GP to accelerate tea breeding.

### Unlocking the potential for tea germplasm for breeding

Evaluation of germplasm provides insights into the evolution and natural selection of tea plants, and identifies valuable breeding materials. Historically, germplasm characterization was a major bottleneck; however, the advantages of GP-based screening and high-throughput phenotyping (HTP) have introduced rapid and cost-effective methods to address these challenges. Among HTP techniques, hyperspectral imaging has been widely applied in tea phenotyping. Wang *et al.* (2018) and Yamashita *et al.* (2020a) used hyperspectral imaging to estimate nitrogen content in tea leaves, demonstrating its potential as an indicator for growth management (Wang *et al.* 2018, Yamashita *et al.* 2020a). Tu *et al.* (2018) and Yamashita *et al.* (2021b) developed a method to estimate the contents of tea quality-related metabolites in tea leaves (Tu *et al.* 2018, Yamashita *et al.* 2021b). These studies were conducted using data from a single cultivar under different cultivation conditions, which restricts their broader applicability to other cultivars. Chen *et al.* (2021) proposed a model for evaluating drought tolerance in tea germplasm by analyzing the relationships between hyperspectral data and drought-related phenotypes in 10 cultivars (Chen *et al.* 2021).

The HTP technology will accelerate the characterization of germplasm and enhance their utilization in breeding programs. Furthermore, integrating HTP-derived phenotypic data with GEBVs enables precise, data-driven selection of promising accessions, paving the way for efficient breeding pipelines. Models trained on phenotypic and genotypic data from training population can generate GEBVs for untested but genotyped accessions using GP. Such approaches have been successfully applied to explore genetic diversity in crop gene banks, including sorghum (Yu *et al.* 2016), wheat (Crossa *et al.* 2016), and soybean (Jarquin *et al.* 2016). Zhou *et al.* (2023) further demonstrated that incorporating GEBVs generated by GP enhanced SNP detection signals in a GWAS for nitrogen use efficiency in *Populus cathayana* (Zhou *et al.* 2023). These findings highlight that integrating GP with germplasm screening not only facilitates the effective use of genetic resources but also accelerates the discovery of trait-associated candidate genes.

Core collections, which aim to capture the maximum possible genetic diversity of a species within a manageable subset of accessions, are valuable resources for purposes such as conservation, genetic research, and breeding (Brown 1989, Odong *et al.* 2013). These collections allow researchers and breeders to utilize a representative accession of the entire germplasm without the need for extensive screening. In Japan, approximately 7,800 accessions collected from 14 countries are preserved at the NARO Institute of Vegetable and Tea Science (NIVTS), Japan (Takeda 2002). Taniguchi *et al.* (2014) analyzed 788 of these accessions using 23 SSR markers, and developed a core collection comprising 192 accessions, which captured 99.5% of alleles present in the 788 accessions (Taniguchi *et al.* 2014). But, core collections may not always be sufficient for targeting specific traits (Brown and Spillane 1999). In addition, key accessions can be overlooked during their construction (Brown 1995). Therefore, breeders should exercise caution when developing and applying core collections in breeding programs. These challenges highlight the need for next-generation germplasm management strategies that integrate genomic tools, advanced phenotyping, and data-driven approaches.

## Conclusion

Tea breeders in Japan have relied on traditional methods to develop key modern cultivars. Tea breeding is now undergoing a transition driven by advances in modern technologies. To further enhance tea production and consumption, it is essential to develop new cultivars and improve breeding efficiency. Genomics-based breeding offers the potential to accelerate selection processes and enable more effective utilization of available tea germplasm. Future tea breeding programs will increasingly incorporate genomics, phenomics, and data science to develop cultivars with greater climate resilience and to fulfill global health-related objectives.

## Author Contribution Statement

YI and HY wrote the original manuscript. TI reviewed and edited the manuscript. All authors read and approved the manuscript. YI and HY have contributed equally to the overall supervision, conceptual design, and coordination of this review article.

## Figures and Tables

**Table 1.** Summary of representative reference genomes in tea plants

Cultivars	Varieties	Types	Genome size (Gb)	N50 of scaffolds (Mb)	BUSCO score (Genome,%)	Number of genes (loci)	References
Yunkang10	var. *assamica*	Scaffold-scale	3.00	0.45	85.20	36,951	Xia *et al.* 2017
Shuchazao	var. *sinensis*	Scaffold-scale	2.89	1.39	91.40	33,932	Wei *et al.* 2018
Shuchazao	var. *sinensis*	Chromosome-scale	2.94	167.10	90.60	50,525	Xia *et al.* 2020
Longjing43	var. *sinensis*	Chromosome-scale	3.26	143.85	88.36	33,556	Wang *et al.* 2020
Tieguanyin	var. *sinensis*	Chromosome-scale	3.06	213.00	93.70	42,825	Zhang *et al.* 2021
Tieguanyin	var. *sinensis*	HapA	3.06	No data	84.80	42,628	Zhang *et al.* 2021
Tieguanyin	var. *sinensis*	HapB	2.92	No data	83.20	42,628	Zhang *et al.* 2021
Huangdan	var. *sinensis*	Chromosome-scale	2.94	213.00	95.00	43,779	Wang *et al.* 2021
Seimei	var. *sinensis*	Chromosome-scale	3.20	214.90	94.80	55,235	Kawahara *et al.* 2024
Zijuan	var. *assamica*	Chromosome-scale	3.06	212.00	98.93	40,749	Tariq *et al.* 2024
Anjibaicha	var. *sinensis*	Chromosome-scale	3.24	202.00	99.14	41,429	Tariq *et al.* 2024
L618	Wild tea plant	Chromosome-scale	3.01	198.00	99.05	41,401	Tariq *et al.* 2024
